# Crystal structure of 2-butyl­sulfanyl-4,6-bis­[(*E*)-4-(di­methyl­amino)­styr­yl]pyrimidine

**DOI:** 10.1107/S2056989015021441

**Published:** 2015-11-21

**Authors:** Jingbao Song, Qiang Zhou, Aijian Wang

**Affiliations:** a China-Australia Joint Research Center for Functional Molecular Materials, Scientific Research Academy, Jiangsu University, Zhenjiang 212013, People’s Republic of China

**Keywords:** crystal structure, pyrimidine, *D–A–D* inter­action

## Abstract

In the title compound, C_28_H_34_N_4_S, the dihedral angles between the pyrimidine ring and the pendant 4-(di­methyl­amino)­benzene rings are 14.20 (5) and 14.56 (4)°. The butyl side chain adopts an *anti* conformation [C—C—C—C = −171.53 (13)°]. No directional inter­actions beyond van der Waals contacts occur in the crystal structure The title mol­ecule has a *D–A–D* structure, in which the pyrimidine ring is the electron-withdrawing part and the 4-(di­methyl­amino)­benzene rings are the electron-donating parts.

## Related literature   

For general background to pyrimidine derivatives and their applications, see: Walker *et al.* (2009 ▸); van Laar *et al.* (2001 ▸); Deng *et al.* (2008 ▸); Nguyen (2008 ▸). For further synthetic details, see: Liu *et al.* (2007 ▸).
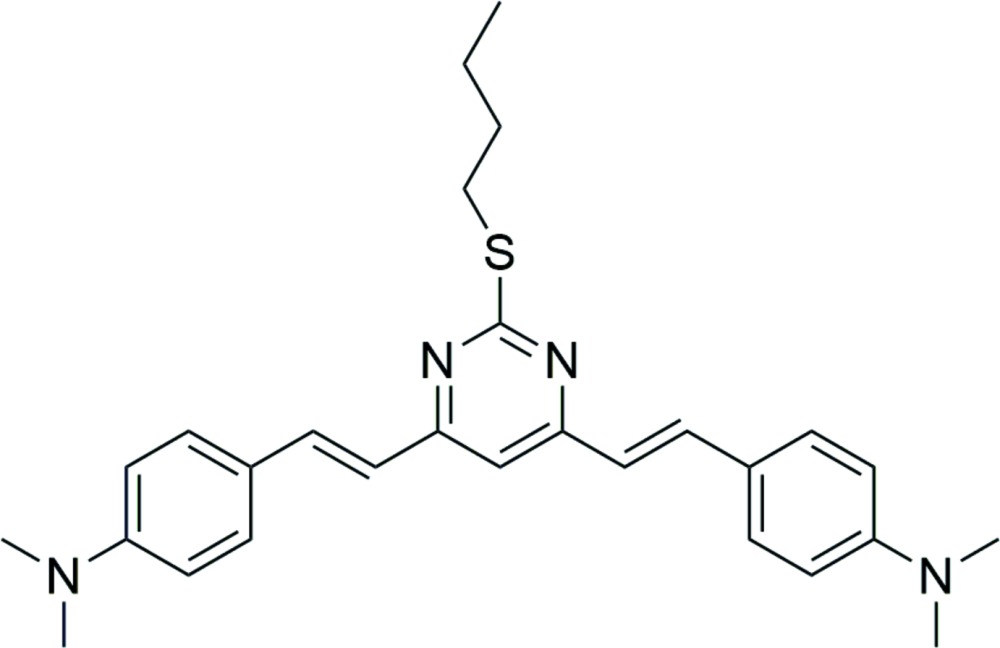


## Experimental   

### Crystal data  

C_28_H_34_N_4_S
*M* *_r_* = 458.65
Monoclinic, 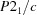

*a* = 7.4425 (15) Å
*b* = 12.583 (3) Å
*c* = 27.448 (6) Å
β = 99.31 (3)°
*V* = 2536.6 (10) Å^3^
*Z* = 4
Mo *K*α radiation
μ = 0.15 mm^−1^
*T* = 293 K
0.20 × 0.20 × 0.20 mm

### Data collection  

Rigaku Saturn724+ CCD diffractometer
Absorption correction: multi-scan *CrystalClear*; Rigaku, 2008 ▸) *T*_min_ = 0.795, *T*_max_ = 1.000
12427 measured reflections
4822 independent reflections
4371 reflections with *I* > 2σ(*I*)
*R*_int_ = 0.021

### Refinement  

*R*[*F* ^2^ > 2σ(*F* ^2^)] = 0.041
*wR*(*F* ^2^) = 0.104
*S* = 1.07
4822 reflections
303 parameters
H-atom parameters constrained
Δρ_max_ = 0.17 e Å^−3^
Δρ_min_ = −0.29 e Å^−3^

### 

Data collection: *CrystalClear* (Rigaku, 2008 ▸); cell refinement: *CrystalClear*; data reduction: *CrystalClear*; program(s) used to solve structure: *SHELXTL* (Sheldrick, 2008 ▸); program(s) used to refine structure: *SHELXL2014* (Sheldrick, 2015 ▸); molecular graphics: *SHELXTL*; software used to prepare material for publication: *SHELXTL*.

## Supplementary Material

Crystal structure: contains datablock(s) I, New_Global_Publ_Block. DOI: 10.1107/S2056989015021441/hb7541sup1.cif

Structure factors: contains datablock(s) I. DOI: 10.1107/S2056989015021441/hb7541Isup2.hkl

Supporting information file. DOI: 10.1107/S2056989015021441/hb7541Isup3.docx

Supporting information file. DOI: 10.1107/S2056989015021441/hb7541Isup4.cdx

Supporting information file. DOI: 10.1107/S2056989015021441/hb7541Isup5.cml

**Fig. 1.**
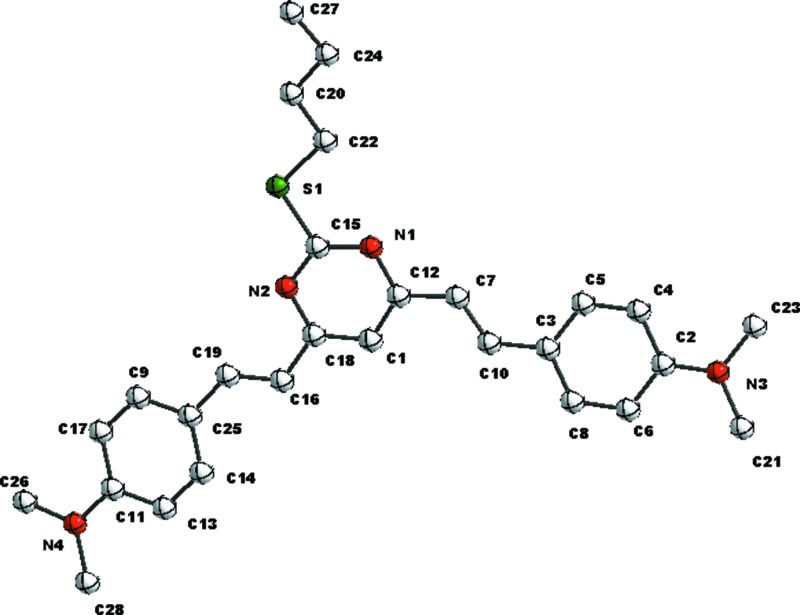
The title compound.

. DOI: 10.1107/S2056989015021441/hb7541fig1.tif
The title compound.

CCDC reference: 1435236

Additional supporting information:  crystallographic information; 3D view; checkCIF report

## supplementary crystallographic information

### Crystal data

**Table:**

C_28_H_34_N_4_S	*F*(000) = 984
*M**_r_* = 458.65	*D*_x_ = 1.201 Mg m^−^^3^
Monoclinic, *P*2_1_/*c*	Mo *K*α radiation, λ = 0.71073 Å
*a* = 7.4425 (15) Å	Cell parameters from 8318 reflections
*b* = 12.583 (3) Å	θ = 3.9–28.7°
*c* = 27.448 (6) Å	µ = 0.15 mm^−^^1^
β = 99.31 (3)°	*T* = 293 K
*V* = 2536.6 (10) Å^3^	Prism, colorless
*Z* = 4	0.20 × 0.20 × 0.20 mm

### Data collection

**Table:**

Rigaku Saturn724+ CCD diffractometer	4371 reflections with *I* > 2σ(*I*)
Detector resolution: 28.5714 pixels mm^-1^	*R*_int_ = 0.021
dtprofit.ref scans	θ_max_ = 26.0°, θ_min_ = 4.0°
Absorption correction: multi-scan *CrystalClear*; Rigaku, 2008)	*h* = −8→9
*T*_min_ = 0.795, *T*_max_ = 1.000	*k* = −14→15
12427 measured reflections	*l* = −32→21
4822 independent reflections	

### Refinement

**Table:**

Refinement on *F*^2^	0 restraints
Least-squares matrix: full	Hydrogen site location: inferred from neighbouring sites
*R*[*F*^2^ > 2σ(*F*^2^)] = 0.041	H-atom parameters constrained
*wR*(*F*^2^) = 0.104	*w* = 1/[σ^2^(*F*_o_^2^) + (0.0542*P*)^2^ + 0.4771*P*] where *P* = (*F*_o_^2^ + 2*F*_c_^2^)/3
*S* = 1.07	(Δ/σ)_max_ = 0.001
4822 reflections	Δρ_max_ = 0.17 e Å^−^^3^
303 parameters	Δρ_min_ = −0.29 e Å^−^^3^

### Special details

**Table:**

Geometry. All e.s.d.'s (except the e.s.d. in the dihedral angle between two l.s. planes) are estimated using the full covariance matrix. The cell e.s.d.'s are taken into account individually in the estimation of e.s.d.'s in distances, angles and torsion angles; correlations between e.s.d.'s in cell parameters are only used when they are defined by crystal symmetry. An approximate (isotropic) treatment of cell e.s.d.'s is used for estimating e.s.d.'s involving l.s. planes.

### Fractional atomic coordinates and isotropic or equivalent isotropic displacement parameters (Å^2^)

**Table:**

	*x*	*y*	*z*	*U*_iso_*/*U*_eq_	
S1	0.26097 (5)	1.10814 (3)	0.80444 (2)	0.03105 (12)	
N1	0.24791 (15)	0.95402 (9)	0.73631 (4)	0.0268 (3)	
N2	0.35953 (16)	0.91412 (9)	0.82151 (4)	0.0275 (3)	
N3	0.12735 (17)	0.58633 (10)	0.45410 (4)	0.0326 (3)	
N4	0.75955 (18)	0.54619 (10)	1.07267 (5)	0.0361 (3)	
C1	0.34216 (19)	0.77758 (11)	0.76064 (5)	0.0277 (3)	
H1	0.3574	0.7068	0.7525	0.033*	
C2	0.15843 (18)	0.62194 (11)	0.50181 (5)	0.0260 (3)	
C3	0.23233 (18)	0.69834 (11)	0.60088 (5)	0.0258 (3)	
C4	0.08957 (19)	0.72030 (11)	0.51535 (5)	0.0278 (3)	
H4	0.0180	0.7611	0.4915	0.033*	
C5	0.12613 (18)	0.75688 (11)	0.56307 (5)	0.0273 (3)	
H5	0.0793	0.8223	0.5706	0.033*	
C6	0.26230 (19)	0.56204 (11)	0.53969 (5)	0.0275 (3)	
H6	0.3075	0.4960	0.5324	0.033*	
C7	0.23083 (18)	0.82523 (11)	0.67185 (5)	0.0280 (3)	
H7	0.1684	0.8759	0.6509	0.034*	
C8	0.29778 (19)	0.59998 (11)	0.58746 (5)	0.0275 (3)	
H8	0.3676	0.5588	0.6116	0.033*	
C9	0.56244 (18)	0.75441 (11)	0.98657 (5)	0.0278 (3)	
H9	0.5156	0.8226	0.9881	0.033*	
C10	0.27343 (18)	0.73315 (11)	0.65182 (5)	0.0275 (3)	
H10	0.3383	0.6850	0.6736	0.033*	
C11	0.69693 (19)	0.59909 (11)	1.02945 (5)	0.0266 (3)	
C12	0.27576 (18)	0.85130 (11)	0.72417 (5)	0.0259 (3)	
C13	0.70746 (19)	0.55396 (11)	0.98288 (5)	0.0281 (3)	
H13	0.7569	0.4864	0.9812	0.034*	
C14	0.64582 (19)	0.60825 (11)	0.93992 (5)	0.0280 (3)	
H14	0.6542	0.5763	0.9098	0.034*	
C15	0.29105 (18)	0.97763 (11)	0.78419 (5)	0.0263 (3)	
C16	0.46067 (18)	0.73921 (11)	0.84922 (5)	0.0283 (3)	
H16	0.4841	0.6691	0.8416	0.034*	
C17	0.62066 (19)	0.70111 (11)	1.03003 (5)	0.0288 (3)	
H17	0.6094	0.7328	1.0600	0.035*	
C18	0.38519 (18)	0.81161 (11)	0.80933 (5)	0.0262 (3)	
C19	0.49716 (18)	0.77085 (11)	0.89650 (5)	0.0283 (3)	
H19	0.4716	0.8418	0.9021	0.034*	
C20	0.20267 (19)	1.29855 (11)	0.76013 (5)	0.0269 (3)	
H20A	0.3254	1.3142	0.7764	0.032*	
H20B	0.1200	1.3153	0.7830	0.032*	
C21	0.2041 (2)	0.48648 (12)	0.44047 (6)	0.0353 (4)	
H21A	0.3341	0.4882	0.4498	0.053*	
H21B	0.1737	0.4764	0.4054	0.053*	
H21C	0.1553	0.4290	0.4572	0.053*	
C22	0.1885 (2)	1.18094 (11)	0.74790 (5)	0.0301 (3)	
H22A	0.2656	1.1635	0.7237	0.036*	
H22B	0.0639	1.1626	0.7343	0.036*	
C23	0.0143 (2)	0.64497 (13)	0.41538 (6)	0.0393 (4)	
H23A	−0.0994	0.6621	0.4257	0.059*	
H23B	−0.0079	0.6024	0.3860	0.059*	
H23C	0.0754	0.7093	0.4087	0.059*	
C24	0.1577 (2)	1.36854 (12)	0.71461 (6)	0.0316 (3)	
H24A	0.2276	1.3449	0.6897	0.038*	
H24B	0.0296	1.3610	0.7011	0.038*	
C25	0.57083 (18)	0.71020 (11)	0.94023 (5)	0.0257 (3)	
C26	0.7276 (3)	0.58937 (14)	1.11936 (6)	0.0448 (4)	
H26A	0.7862	0.6573	1.1247	0.067*	
H26B	0.7764	0.5419	1.1456	0.067*	
H26C	0.5991	0.5976	1.1188	0.067*	
C27	0.1994 (2)	1.48477 (13)	0.72632 (7)	0.0441 (4)	
H27A	0.1323	1.5080	0.7514	0.066*	
H27B	0.1648	1.5266	0.6971	0.066*	
H27C	0.3274	1.4931	0.7378	0.066*	
C28	0.8384 (2)	0.44130 (12)	1.07180 (6)	0.0378 (4)	
H28A	0.7500	0.3935	1.0544	0.057*	
H28B	0.8745	0.4165	1.1050	0.057*	
H28C	0.9429	0.4443	1.0554	0.057*	

### Atomic displacement parameters (Å^2^)

**Table:**

	*U*^11^	*U*^22^	*U*^33^	*U*^12^	*U*^13^	*U*^23^
S1	0.0411 (2)	0.0249 (2)	0.0253 (2)	0.00081 (15)	0.00000 (16)	−0.00120 (13)
N1	0.0254 (6)	0.0266 (6)	0.0281 (7)	−0.0005 (5)	0.0034 (5)	−0.0013 (5)
N2	0.0277 (6)	0.0266 (6)	0.0277 (6)	−0.0004 (5)	0.0030 (5)	0.0009 (5)
N3	0.0387 (7)	0.0337 (7)	0.0241 (6)	0.0033 (6)	0.0012 (5)	−0.0021 (5)
N4	0.0495 (8)	0.0314 (7)	0.0266 (7)	0.0039 (6)	0.0038 (6)	0.0023 (5)
C1	0.0287 (7)	0.0245 (7)	0.0302 (8)	0.0005 (6)	0.0057 (6)	−0.0008 (6)
C2	0.0244 (7)	0.0293 (7)	0.0247 (7)	−0.0035 (6)	0.0055 (5)	−0.0001 (6)
C3	0.0242 (7)	0.0275 (7)	0.0264 (7)	−0.0011 (6)	0.0058 (5)	0.0011 (6)
C4	0.0279 (7)	0.0279 (7)	0.0271 (7)	0.0022 (6)	0.0030 (6)	0.0052 (6)
C5	0.0289 (7)	0.0242 (7)	0.0293 (8)	0.0019 (6)	0.0060 (6)	0.0007 (6)
C6	0.0284 (7)	0.0250 (7)	0.0299 (8)	0.0015 (6)	0.0067 (6)	−0.0007 (6)
C7	0.0264 (7)	0.0295 (8)	0.0275 (7)	0.0012 (6)	0.0024 (6)	0.0015 (6)
C8	0.0280 (7)	0.0276 (8)	0.0265 (7)	0.0015 (6)	0.0032 (6)	0.0037 (6)
C9	0.0258 (7)	0.0247 (7)	0.0327 (8)	−0.0003 (6)	0.0042 (6)	−0.0036 (6)
C10	0.0251 (7)	0.0298 (8)	0.0273 (7)	−0.0001 (6)	0.0033 (5)	0.0022 (6)
C11	0.0247 (7)	0.0274 (7)	0.0277 (7)	−0.0034 (6)	0.0044 (6)	−0.0005 (6)
C12	0.0206 (7)	0.0286 (7)	0.0289 (8)	−0.0019 (6)	0.0050 (5)	−0.0024 (6)
C13	0.0285 (7)	0.0244 (7)	0.0317 (8)	0.0015 (6)	0.0056 (6)	−0.0020 (6)
C14	0.0282 (7)	0.0306 (8)	0.0255 (7)	0.0007 (6)	0.0057 (6)	−0.0041 (6)
C15	0.0230 (7)	0.0275 (7)	0.0281 (8)	−0.0026 (6)	0.0036 (5)	−0.0010 (6)
C16	0.0288 (7)	0.0269 (7)	0.0296 (8)	0.0003 (6)	0.0061 (6)	0.0017 (6)
C17	0.0309 (7)	0.0293 (8)	0.0268 (8)	−0.0010 (6)	0.0065 (6)	−0.0050 (6)
C18	0.0218 (7)	0.0289 (7)	0.0284 (7)	−0.0011 (6)	0.0060 (5)	0.0014 (6)
C19	0.0247 (7)	0.0278 (7)	0.0325 (8)	0.0001 (6)	0.0051 (6)	0.0009 (6)
C20	0.0264 (7)	0.0271 (7)	0.0269 (7)	0.0007 (6)	0.0038 (6)	−0.0003 (6)
C21	0.0400 (8)	0.0375 (9)	0.0292 (8)	0.0009 (7)	0.0079 (6)	−0.0060 (6)
C22	0.0342 (8)	0.0293 (8)	0.0254 (7)	−0.0024 (6)	0.0002 (6)	−0.0005 (6)
C23	0.0493 (9)	0.0411 (9)	0.0253 (8)	0.0017 (8)	−0.0004 (7)	0.0002 (7)
C24	0.0293 (8)	0.0347 (8)	0.0311 (8)	0.0048 (6)	0.0059 (6)	0.0051 (6)
C25	0.0221 (7)	0.0274 (7)	0.0277 (7)	−0.0020 (6)	0.0043 (5)	−0.0006 (6)
C26	0.0611 (11)	0.0480 (10)	0.0251 (8)	0.0043 (8)	0.0066 (8)	0.0021 (7)
C27	0.0385 (9)	0.0329 (9)	0.0618 (12)	0.0038 (7)	0.0107 (8)	0.0122 (8)
C28	0.0381 (9)	0.0349 (9)	0.0406 (9)	0.0042 (7)	0.0068 (7)	0.0095 (7)

### Geometric parameters (Å, º)

**Table:**

S1—C15	1.7597 (15)	C13—C14	1.376 (2)
S1—C22	1.8076 (15)	C13—H13	0.9300
N1—C15	1.3348 (18)	C14—C25	1.400 (2)
N1—C12	1.3590 (18)	C14—H14	0.9300
N2—C15	1.3339 (18)	C16—C19	1.342 (2)
N2—C18	1.3538 (18)	C16—C18	1.4647 (19)
N3—C2	1.3679 (18)	C16—H16	0.9300
N3—C23	1.4463 (19)	C17—H17	0.9300
N3—C21	1.4539 (19)	C19—C25	1.4529 (19)
N4—C11	1.3742 (18)	C19—H19	0.9300
N4—C28	1.446 (2)	C20—C22	1.517 (2)
N4—C26	1.447 (2)	C20—C24	1.5211 (19)
C1—C18	1.391 (2)	C20—H20A	0.9700
C1—C12	1.395 (2)	C20—H20B	0.9700
C1—H1	0.9300	C21—H21A	0.9600
C2—C6	1.409 (2)	C21—H21B	0.9600
C2—C4	1.412 (2)	C21—H21C	0.9600
C3—C8	1.401 (2)	C22—H22A	0.9700
C3—C5	1.4069 (19)	C22—H22B	0.9700
C3—C10	1.450 (2)	C23—H23A	0.9600
C4—C5	1.373 (2)	C23—H23B	0.9600
C4—H4	0.9300	C23—H23C	0.9600
C5—H5	0.9300	C24—C27	1.519 (2)
C6—C8	1.380 (2)	C24—H24A	0.9700
C6—H6	0.9300	C24—H24B	0.9700
C7—C10	1.342 (2)	C26—H26A	0.9600
C7—C12	1.458 (2)	C26—H26B	0.9600
C7—H7	0.9300	C26—H26C	0.9600
C8—H8	0.9300	C27—H27A	0.9600
C9—C17	1.376 (2)	C27—H27B	0.9600
C9—C25	1.399 (2)	C27—H27C	0.9600
C9—H9	0.9300	C28—H28A	0.9600
C10—H10	0.9300	C28—H28B	0.9600
C11—C17	1.405 (2)	C28—H28C	0.9600
C11—C13	1.413 (2)		
			
C15—S1—C22	103.73 (7)	C11—C17—H17	119.8
C15—N1—C12	115.53 (12)	N2—C18—C1	120.73 (13)
C15—N2—C18	115.65 (12)	N2—C18—C16	117.41 (13)
C2—N3—C23	121.63 (13)	C1—C18—C16	121.86 (13)
C2—N3—C21	121.11 (12)	C16—C19—C25	129.28 (14)
C23—N3—C21	117.25 (12)	C16—C19—H19	115.4
C11—N4—C28	120.65 (13)	C25—C19—H19	115.4
C11—N4—C26	120.10 (13)	C22—C20—C24	112.62 (12)
C28—N4—C26	118.85 (13)	C22—C20—H20A	109.1
C18—C1—C12	118.99 (13)	C24—C20—H20A	109.1
C18—C1—H1	120.5	C22—C20—H20B	109.1
C12—C1—H1	120.5	C24—C20—H20B	109.1
N3—C2—C6	121.25 (13)	H20A—C20—H20B	107.8
N3—C2—C4	121.79 (13)	N3—C21—H21A	109.5
C6—C2—C4	116.97 (13)	N3—C21—H21B	109.5
C8—C3—C5	116.50 (13)	H21A—C21—H21B	109.5
C8—C3—C10	119.50 (13)	N3—C21—H21C	109.5
C5—C3—C10	123.99 (13)	H21A—C21—H21C	109.5
C5—C4—C2	121.37 (13)	H21B—C21—H21C	109.5
C5—C4—H4	119.3	C20—C22—S1	107.68 (10)
C2—C4—H4	119.3	C20—C22—H22A	110.2
C4—C5—C3	121.95 (13)	S1—C22—H22A	110.2
C4—C5—H5	119.0	C20—C22—H22B	110.2
C3—C5—H5	119.0	S1—C22—H22B	110.2
C8—C6—C2	120.91 (13)	H22A—C22—H22B	108.5
C8—C6—H6	119.5	N3—C23—H23A	109.5
C2—C6—H6	119.5	N3—C23—H23B	109.5
C10—C7—C12	124.45 (13)	H23A—C23—H23B	109.5
C10—C7—H7	117.8	N3—C23—H23C	109.5
C12—C7—H7	117.8	H23A—C23—H23C	109.5
C6—C8—C3	122.29 (13)	H23B—C23—H23C	109.5
C6—C8—H8	118.9	C27—C24—C20	111.93 (13)
C3—C8—H8	118.9	C27—C24—H24A	109.2
C17—C9—C25	122.70 (13)	C20—C24—H24A	109.2
C17—C9—H9	118.6	C27—C24—H24B	109.2
C25—C9—H9	118.6	C20—C24—H24B	109.2
C7—C10—C3	128.96 (13)	H24A—C24—H24B	107.9
C7—C10—H10	115.5	C9—C25—C14	116.53 (13)
C3—C10—H10	115.5	C9—C25—C19	118.42 (13)
N4—C11—C17	120.94 (13)	C14—C25—C19	125.02 (13)
N4—C11—C13	121.72 (13)	N4—C26—H26A	109.5
C17—C11—C13	117.34 (13)	N4—C26—H26B	109.5
N1—C12—C1	120.46 (13)	H26A—C26—H26B	109.5
N1—C12—C7	115.82 (12)	N4—C26—H26C	109.5
C1—C12—C7	123.72 (13)	H26A—C26—H26C	109.5
C14—C13—C11	121.09 (13)	H26B—C26—H26C	109.5
C14—C13—H13	119.5	C24—C27—H27A	109.5
C11—C13—H13	119.5	C24—C27—H27B	109.5
C13—C14—C25	121.86 (13)	H27A—C27—H27B	109.5
C13—C14—H14	119.1	C24—C27—H27C	109.5
C25—C14—H14	119.1	H27A—C27—H27C	109.5
N2—C15—N1	128.61 (13)	H27B—C27—H27C	109.5
N2—C15—S1	111.65 (10)	N4—C28—H28A	109.5
N1—C15—S1	119.74 (11)	N4—C28—H28B	109.5
C19—C16—C18	122.09 (13)	H28A—C28—H28B	109.5
C19—C16—H16	119.0	N4—C28—H28C	109.5
C18—C16—H16	119.0	H28A—C28—H28C	109.5
C9—C17—C11	120.46 (13)	H28B—C28—H28C	109.5
C9—C17—H17	119.8		
			
C23—N3—C2—C6	177.24 (14)	C17—C11—C13—C14	0.2 (2)
C21—N3—C2—C6	−1.5 (2)	C11—C13—C14—C25	0.2 (2)
C23—N3—C2—C4	−3.2 (2)	C18—N2—C15—N1	−1.5 (2)
C21—N3—C2—C4	177.99 (13)	C18—N2—C15—S1	179.25 (10)
N3—C2—C4—C5	−177.99 (13)	C12—N1—C15—N2	0.8 (2)
C6—C2—C4—C5	1.5 (2)	C12—N1—C15—S1	180.00 (10)
C2—C4—C5—C3	−0.5 (2)	C22—S1—C15—N2	174.93 (10)
C8—C3—C5—C4	−0.6 (2)	C22—S1—C15—N1	−4.41 (13)
C10—C3—C5—C4	−179.48 (13)	C25—C9—C17—C11	1.8 (2)
N3—C2—C6—C8	177.99 (13)	N4—C11—C17—C9	178.41 (13)
C4—C2—C6—C8	−1.5 (2)	C13—C11—C17—C9	−1.2 (2)
C2—C6—C8—C3	0.5 (2)	C15—N2—C18—C1	0.26 (19)
C5—C3—C8—C6	0.6 (2)	C15—N2—C18—C16	179.83 (12)
C10—C3—C8—C6	179.53 (13)	C12—C1—C18—N2	1.4 (2)
C12—C7—C10—C3	178.55 (13)	C12—C1—C18—C16	−178.11 (12)
C8—C3—C10—C7	177.03 (14)	C19—C16—C18—N2	2.6 (2)
C5—C3—C10—C7	−4.1 (2)	C19—C16—C18—C1	−177.88 (13)
C28—N4—C11—C17	−179.83 (13)	C18—C16—C19—C25	179.77 (13)
C26—N4—C11—C17	7.6 (2)	C24—C20—C22—S1	175.96 (10)
C28—N4—C11—C13	−0.3 (2)	C15—S1—C22—C20	−166.56 (10)
C26—N4—C11—C13	−172.87 (14)	C22—C20—C24—C27	−171.53 (13)
C15—N1—C12—C1	1.11 (19)	C17—C9—C25—C14	−1.3 (2)
C15—N1—C12—C7	−179.34 (12)	C17—C9—C25—C19	176.85 (13)
C18—C1—C12—N1	−2.2 (2)	C13—C14—C25—C9	0.3 (2)
C18—C1—C12—C7	178.32 (13)	C13—C14—C25—C19	−177.72 (13)
C10—C7—C12—N1	169.79 (13)	C16—C19—C25—C9	−167.16 (14)
C10—C7—C12—C1	−10.7 (2)	C16—C19—C25—C14	10.8 (2)
N4—C11—C13—C14	−179.37 (13)		
