# Supplementary material for: Crystal structure of 2-butyl­sulfanyl-4,6-bis­[(E)-4-(di­methyl­amino)­styr­yl]pyrimidine
Source: Acta Crystallogr E Crystallogr Commun. 2015 Nov 21;71(Pt 12):o978. doi: 10.1107/S2056989015021441 (PMC4719932; doi:10.1107/S2056989015021441)
Supplement: Supplementary file 3 [file e-71-0o978-Isup3.docx]

**Crystal structure of 4,6-bis[(E)-4-dimethylamino-styryl]-2-butylsulfanyl) pyrimidine**

**Jingbao Song, Qiang Zhou and Aijian Wang***

I

**S1. Introduction**

**S2. Experimental**

**S2.1. Synthesis and crystallization**

2-(butylthio)-4,6-dimethylpyrimidine (2.00g, 10.19mmol) and 4-(dimethylamino)benzaldehyde(3.2g, 21.45mmol) were added in an aqueous solution of sodium hydroxide(5M, 30 ml) containing tetrabutylammonium iodide(10 mol% versus the heterocycle) and mixed. The mixture was heated under reflux for 5h. After cooling, the reaction mixture was extracted with dichloromethane (100ml*4). The extract solution was dried with magnesium sulfate. After removal of the drying agent by filtration, the solvent was removed by evaporation under reduced pressure. The crude product was recrystallized to afford yellow prisms of the title compound, (I) (3.51g, yield 74.9%).

**S2.2. Refinement**

**S3. Results and discussion**


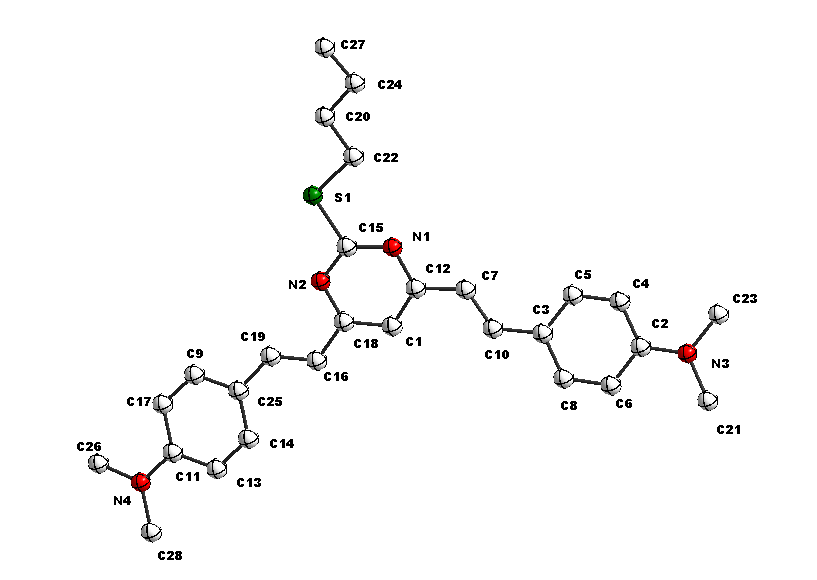


**Figure 1**

The molecular structure of (I) showing 50% displacement ellipsoids.

**4,6-bis[(E)-4-dimethylamino-styryl]-2-butylsulfanyl) pyrimidine**

**Crystal data**

C28H34N4S

Mr=458.65 F(000)=984

Monoclinic, P 2_1/c_ D_X_=1.201mg.m^-3^

*a*=7.4425(15) Å Mo Kα radiation, *λ* = 0.71073 Å

*b*=12.583(3) Å Cell parameters from 4822 reflections

*c*=27.448(6) Å θ = 23.948–26.028°

*α=*90° µ = 0.150 mm ^−1^

*β=*99.31（3）° T = 293(2) K

*γ=*90°

V=2536.6(10) Å^3^

Z=4

**Data collection**

Rigaku Saturn724+ 12427 measured reflections

Diffractometer 4822 independent reflections

Radiation source: fine-focus sealed tube R (int)= 0.0214

Graphite monochromator *θ* max = 26.028°, *θ* min = 3.948°

*ω* scans

Absorption correction: multi-scan *h* = −8→9

(CrystalClear; Rigaku, 2008)  *k* = −14→15

T _min_ = 0.7946, T _max_ = 1.000  *l* = −32→21

**Refinement**

Refinement on *F*^2^

Least-squares matrix: full Secondary atom site location: difference Fourier map

R[*F*^2^ > *2σ(F^2^ )*] = 0.0408 Hydrogen site location: inferred from neighbouring sites

*w*R(*F^2^* ) = 0.1006 H-atom parameters constrained

0 restraints *w* = 1/[*σ*^2^ *(F_o_*^2^ ) + (0.0344P)^2^ + 0.2887P]

4822 reflections where P = (*F_o_*^2^ + 2F_c_^2^ )/3

303 parameters Δρ _min_ = −0.290 e Å^−3^

Primary atom site location: Δρ _max_ = 0.173 e Å^−3^

structure-invariantdirect methods

**Special details**

**Geometry.** All e.s.d.’s (except the e.s.d. in the dihedral angle between two l.s. planes) are estimated using full covariance matrix. The cell e.s.d.’s are taken into account individually in the estimation of e.s.d.’s in distances, angles and torsion angles; correlations between e.s.d.’s in cell parameters are only used when they are defined by crystal symmetry. An approximate (isotropic) treatment of cell e.s.d.’s is used for estimating e.s.d.’s involving l.s. planes.

**Refinement.** Refinement of F^2^ against ALL reflections. The weighted R-factor *w*R and goodness of fit *S* are based on *F*^2^, concentional R-factors R are based on *F*, with *F* set to zero for negative *F*^2^. The shreshold expression of F^2^>σ(F^2^) is used only for calculating R-factors(gt) etc, and is not relevant to the choice of reflections for refinement. R-factors based on F^2^ are statistically about twice as large as those based on F, and R- factors based on ALL data will be even larger.

Atomic coordinates ( x10^4^) and equivalent isotropic displacement parameters (A^2^ x 10^3^)

U(eq) is defined as one third of the trace of the orthogonalized Uij tensor.

________________________________________________________________

x y z U(eq)

________________________________________________________________

S(1) 2610(1) 11081(1) 8044(1) 31(1)

N(1) 2479(2) 9540(1) 7363(1) 27(1)

N(2) 3595(2) 9141(1) 8215(1) 28(1)

N(3) 1274(2) 5863(1) 4541(1) 33(1)

N(4) 7596(2) 5462(1) 10727(1) 36(1)

C(1) 3422(2) 7776(1) 7606(1) 28(1)

C(2) 1584(2) 6219(1) 5018(1) 26(1)

C(3) 2323(2) 6983(1) 6009(1) 26(1)

C(4) 896(2) 7203(1) 5154(1) 28(1)

C(5) 1261(2) 7569(1) 5631(1) 27(1)

C(6) 2623(2) 5620(1) 5397(1) 28(1)

C(7) 2308(2) 8252(1) 6718(1) 28(1)

C(8) 2978(2) 6000(1) 5875(1) 28(1)

C(9) 5624(2) 7544(1) 9866(1) 28(1)

C(10) 2734(2) 7332(1) 6518(1) 28(1)

C(11) 6969(2) 5991(1) 10295(1) 27(1)

C(12) 2758(2) 8513(1) 7242(1) 26(1)

C(13) 7075(2) 5540(1) 9829(1) 28(1)

C(14) 6458(2) 6083(1) 9399(1) 28(1)

C(15) 2910(2) 9776(1) 7842(1) 26(1)

C(16) 4607(2) 7392(1) 8492(1) 28(1)

C(17) 6207(2) 7011(1) 10300(1) 29(1)

C(18) 3852(2) 8116(1) 8093(1) 26(1)

C(19) 4972(2) 7708(1) 8965(1) 28(1)

C(20) 2027(2) 12986(1) 7601(1) 27(1)

C(21) 2041(2) 4865(1) 4405(1) 35(1)

C(22) 1885(2) 11809(1) 7479(1) 30(1)

C(23) 143(2) 6450(1) 4154(1) 39(1)

C(24) 1577(2) 13685(1) 7146(1) 32(1)

C(25) 5708(2) 7102(1) 9402(1) 26(1)

C(26) 7276(3) 5894(1) 11194(1) 45(1)

C(27) 1994(2) 14848(1) 7263(1) 44(1)

C(28) 8384(2) 4413(1) 10718(1) 38(1)

________________________________________________________________

Bond lengths [Å] and angles [°]

____________________________________________________________

S(1)-C(15) 1.7597(15)

S(1)-C(22) 1.8076(15)

N(1)-C(15) 1.3348(18)

N(1)-C(12) 1.3590(18)

N(2)-C(15) 1.3339(18)

N(2)-C(18) 1.3538(18)

N(3)-C(2) 1.3679(18)

N(3)-C(23) 1.4463(19)

N(3)-C(21) 1.4539(19)

N(4)-C(11) 1.3742(18)

N(4)-C(28) 1.446(2)

N(4)-C(26) 1.447(2)

C(1)-C(18) 1.391(2)

C(1)-C(12) 1.395(2)

C(1)-H(1) 0.9300

C(2)-C(6) 1.409(2)

C(2)-C(4) 1.412(2)

C(3)-C(8) 1.401(2)

C(3)-C(5) 1.4069(19)

C(3)-C(10) 1.450(2)

C(4)-C(5) 1.373(2)

C(4)-H(4) 0.9300

C(5)-H(5) 0.9300

C(6)-C(8) 1.380(2)

C(6)-H(6) 0.9300

C(7)-C(10) 1.342(2)

C(7)-C(12) 1.458(2)

C(7)-H(7) 0.9300

C(8)-H(8) 0.9300

C(9)-C(17) 1.376(2)

C(9)-C(25) 1.399(2)

C(9)-H(9) 0.9300

C(10)-H(10) 0.9300

C(11)-C(17) 1.405(2)

C(11)-C(13) 1.413(2)

C(13)-C(14) 1.376(2)

C(13)-H(13) 0.9300

C(14)-C(25) 1.400(2)

C(14)-H(14) 0.9300

C(16)-C(19) 1.342(2)

C(16)-C(18) 1.4647(19)

C(16)-H(16) 0.9300

C(17)-H(17) 0.9300

C(19)-C(25) 1.4529(19)

C(19)-H(19) 0.9300

C(20)-C(22) 1.517(2)

C(20)-C(24) 1.5211(19)

C(20)-H(20A) 0.9700

C(20)-H(20B) 0.9700

C(21)-H(21A) 0.9600

C(21)-H(21B) 0.9600

C(21)-H(21C) 0.9600

C(22)-H(22A) 0.9700

C(22)-H(22B) 0.9700

C(23)-H(23A) 0.9600

C(23)-H(23B) 0.9600

C(23)-H(23C) 0.9600

C(24)-C(27) 1.519(2)

C(24)-H(24A) 0.9700

C(24)-H(24B) 0.9700

C(26)-H(26A) 0.9600

C(26)-H(26B) 0.9600

C(26)-H(26C) 0.9600

C(27)-H(27A) 0.9600

C(27)-H(27B) 0.9600

C(27)-H(27C) 0.9600

C(28)-H(28A) 0.9600

C(28)-H(28B) 0.9600

C(28)-H(28C) 0.9600

C(15)-S(1)-C(22) 103.73(7)

C(15)-N(1)-C(12) 115.53(12)

C(15)-N(2)-C(18) 115.65(12)

C(2)-N(3)-C(23) 121.63(13)

C(2)-N(3)-C(21) 121.11(12)

C(23)-N(3)-C(21) 117.25(12)

C(11)-N(4)-C(28) 120.65(13)

C(11)-N(4)-C(26) 120.10(13)

C(28)-N(4)-C(26) 118.85(13)

C(18)-C(1)-C(12) 118.99(13)

C(18)-C(1)-H(1) 120.5

C(12)-C(1)-H(1) 120.5

N(3)-C(2)-C(6) 121.25(13)

N(3)-C(2)-C(4) 121.79(13)

C(6)-C(2)-C(4) 116.97(13)

C(8)-C(3)-C(5) 116.50(13)

C(8)-C(3)-C(10) 119.50(13)

C(5)-C(3)-C(10) 123.99(13)

C(5)-C(4)-C(2) 121.37(13)

C(5)-C(4)-H(4) 119.3

C(2)-C(4)-H(4) 119.3

C(4)-C(5)-C(3) 121.95(13)

C(4)-C(5)-H(5) 119.0

C(3)-C(5)-H(5) 119.0

C(8)-C(6)-C(2) 120.91(13)

C(8)-C(6)-H(6) 119.5

C(2)-C(6)-H(6) 119.5

C(10)-C(7)-C(12) 124.45(13)

C(10)-C(7)-H(7) 117.8

C(12)-C(7)-H(7) 117.8

C(6)-C(8)-C(3) 122.29(13)

C(6)-C(8)-H(8) 118.9

C(3)-C(8)-H(8) 118.9

C(17)-C(9)-C(25) 122.70(13)

C(17)-C(9)-H(9) 118.6

C(25)-C(9)-H(9) 118.6

C(7)-C(10)-C(3) 128.96(13)

C(7)-C(10)-H(10) 115.5

C(3)-C(10)-H(10) 115.5

N(4)-C(11)-C(17) 120.94(13)

N(4)-C(11)-C(13) 121.72(13)

C(17)-C(11)-C(13) 117.34(13)

N(1)-C(12)-C(1) 120.46(13)

N(1)-C(12)-C(7) 115.82(12)

C(1)-C(12)-C(7) 123.72(13)

C(14)-C(13)-C(11) 121.09(13)

C(14)-C(13)-H(13) 119.5

C(11)-C(13)-H(13) 119.5

C(13)-C(14)-C(25) 121.86(13)

C(13)-C(14)-H(14) 119.1

C(25)-C(14)-H(14) 119.1

N(2)-C(15)-N(1) 128.61(13)

N(2)-C(15)-S(1) 111.65(10)

N(1)-C(15)-S(1) 119.74(11)

C(19)-C(16)-C(18) 122.09(13)

C(19)-C(16)-H(16) 119.0

C(18)-C(16)-H(16) 119.0

C(9)-C(17)-C(11) 120.46(13)

C(9)-C(17)-H(17) 119.8

C(11)-C(17)-H(17) 119.8

N(2)-C(18)-C(1) 120.73(13)

N(2)-C(18)-C(16) 117.41(13)

C(1)-C(18)-C(16) 121.86(13)

C(16)-C(19)-C(25) 129.28(14)

C(16)-C(19)-H(19) 115.4

C(25)-C(19)-H(19) 115.4

C(22)-C(20)-C(24) 112.62(12)

C(22)-C(20)-H(20A) 109.1

C(24)-C(20)-H(20A) 109.1

C(22)-C(20)-H(20B) 109.1

C(24)-C(20)-H(20B) 109.1

H(20A)-C(20)-H(20B) 107.8

N(3)-C(21)-H(21A) 109.5

N(3)-C(21)-H(21B) 109.5

H(21A)-C(21)-H(21B) 109.5

N(3)-C(21)-H(21C) 109.5

H(21A)-C(21)-H(21C) 109.5

H(21B)-C(21)-H(21C) 109.5

C(20)-C(22)-S(1) 107.68(10)

C(20)-C(22)-H(22A) 110.2

S(1)-C(22)-H(22A) 110.2

C(20)-C(22)-H(22B) 110.2

S(1)-C(22)-H(22B) 110.2

H(22A)-C(22)-H(22B) 108.5

N(3)-C(23)-H(23A) 109.5

N(3)-C(23)-H(23B) 109.5

H(23A)-C(23)-H(23B) 109.5

N(3)-C(23)-H(23C) 109.5

H(23A)-C(23)-H(23C) 109.5

H(23B)-C(23)-H(23C) 109.5

C(27)-C(24)-C(20) 111.93(13)

C(27)-C(24)-H(24A) 109.2

C(20)-C(24)-H(24A) 109.2

C(27)-C(24)-H(24B) 109.2

C(20)-C(24)-H(24B) 109.2

H(24A)-C(24)-H(24B) 107.9

C(9)-C(25)-C(14) 116.53(13)

C(9)-C(25)-C(19) 118.42(13)

C(14)-C(25)-C(19) 125.02(13)

N(4)-C(26)-H(26A) 109.5

N(4)-C(26)-H(26B) 109.5

H(26A)-C(26)-H(26B) 109.5

N(4)-C(26)-H(26C) 109.5

H(26A)-C(26)-H(26C) 109.5

H(26B)-C(26)-H(26C) 109.5

C(24)-C(27)-H(27A) 109.5

C(24)-C(27)-H(27B) 109.5

H(27A)-C(27)-H(27B) 109.5

C(24)-C(27)-H(27C) 109.5

H(27A)-C(27)-H(27C) 109.5

H(27B)-C(27)-H(27C) 109.5

N(4)-C(28)-H(28A) 109.5

N(4)-C(28)-H(28B) 109.5

H(28A)-C(28)-H(28B) 109.5

N(4)-C(28)-H(28C) 109.5

H(28A)-C(28)-H(28C) 109.5

H(28B)-C(28)-H(28C) 109.5

_____________________________________________________________

Torsion angles [°] .

________________________________________________________________

C(23)-N(3)-C(2)-C(6) 177.24(14)

C(21)-N(3)-C(2)-C(6) -1.5(2)

C(23)-N(3)-C(2)-C(4) -3.2(2)

C(21)-N(3)-C(2)-C(4) 177.99(13)

N(3)-C(2)-C(4)-C(5) -177.99(13)

C(6)-C(2)-C(4)-C(5) 1.5(2)

C(2)-C(4)-C(5)-C(3) -0.5(2)

C(8)-C(3)-C(5)-C(4) -0.6(2)

C(10)-C(3)-C(5)-C(4) -179.48(13)

N(3)-C(2)-C(6)-C(8) 177.99(13)

C(4)-C(2)-C(6)-C(8) -1.5(2)

C(2)-C(6)-C(8)-C(3) 0.5(2)

C(5)-C(3)-C(8)-C(6) 0.6(2)

C(10)-C(3)-C(8)-C(6) 179.53(13)

C(12)-C(7)-C(10)-C(3) 178.55(13)

C(8)-C(3)-C(10)-C(7) 177.03(14)

C(5)-C(3)-C(10)-C(7) -4.1(2)

C(28)-N(4)-C(11)-C(17) -179.83(13)

C(26)-N(4)-C(11)-C(17) 7.6(2)

C(28)-N(4)-C(11)-C(13) -0.3(2)

C(26)-N(4)-C(11)-C(13) -172.87(14)

C(15)-N(1)-C(12)-C(1) 1.11(19)

C(15)-N(1)-C(12)-C(7) -179.34(12)

C(18)-C(1)-C(12)-N(1) -2.2(2)

C(18)-C(1)-C(12)-C(7) 178.32(13)

C(10)-C(7)-C(12)-N(1) 169.79(13)

C(10)-C(7)-C(12)-C(1) -10.7(2)

N(4)-C(11)-C(13)-C(14) -179.37(13)

C(17)-C(11)-C(13)-C(14) 0.2(2)

C(11)-C(13)-C(14)-C(25) 0.2(2)

C(18)-N(2)-C(15)-N(1) -1.5(2)

C(18)-N(2)-C(15)-S(1) 179.25(10)

C(12)-N(1)-C(15)-N(2) 0.8(2)

C(12)-N(1)-C(15)-S(1) 180.00(10)

C(22)-S(1)-C(15)-N(2) 174.93(10)

C(22)-S(1)-C(15)-N(1) -4.41(13)

C(25)-C(9)-C(17)-C(11) 1.8(2)

N(4)-C(11)-C(17)-C(9) 178.41(13)

C(13)-C(11)-C(17)-C(9) -1.2(2)

C(15)-N(2)-C(18)-C(1) 0.26(19)

C(15)-N(2)-C(18)-C(16) 179.83(12)

C(12)-C(1)-C(18)-N(2) 1.4(2)

C(12)-C(1)-C(18)-C(16) -178.11(12)

C(19)-C(16)-C(18)-N(2) 2.6(2)

C(19)-C(16)-C(18)-C(1) -177.88(13)

C(18)-C(16)-C(19)-C(25) 179.77(13)

C(24)-C(20)-C(22)-S(1) 175.96(10)

C(15)-S(1)-C(22)-C(20) -166.56(10)

C(22)-C(20)-C(24)-C(27) -171.53(13)

C(17)-C(9)-C(25)-C(14) -1.3(2)

C(17)-C(9)-C(25)-C(19) 176.85(13)

C(13)-C(14)-C(25)-C(9) 0.3(2)

C(13)-C(14)-C(25)-C(19) -177.72(13)

C(16)-C(19)-C(25)-C(9) -167.16(14)

C(16)-C(19)-C(25)-C(14) 10.8(2)

________________________________________________________________

Anisotropic displacement parameters (Å^2^ x 10^3^)

_______________________________________________________________________

U11 U22 U33 U23 U13 U12

_______________________________________________________________________

S(1) 41(1) 25(1) 25(1) -1(1) 0(1) 1(1)

N(1) 25(1) 27(1) 28(1) -1(1) 3(1) -1(1)

N(2) 28(1) 27(1) 28(1) 1(1) 3(1) 0(1)

N(3) 39(1) 34(1) 24(1) -2(1) 1(1) 3(1)

N(4) 50(1) 31(1) 27(1) 2(1) 4(1) 4(1)

C(1) 29(1) 24(1) 30(1) -1(1) 6(1) 1(1)

C(2) 24(1) 29(1) 25(1) 0(1) 6(1) -4(1)

C(3) 24(1) 28(1) 26(1) 1(1) 6(1) -1(1)

C(4) 28(1) 28(1) 27(1) 5(1) 3(1) 2(1)

C(5) 29(1) 24(1) 29(1) 1(1) 6(1) 2(1)

C(6) 28(1) 25(1) 30(1) -1(1) 7(1) 2(1)

C(7) 26(1) 30(1) 28(1) 2(1) 2(1) 1(1)

C(8) 28(1) 28(1) 26(1) 4(1) 3(1) 2(1)

C(9) 26(1) 25(1) 33(1) -4(1) 4(1) 0(1)

C(10) 25(1) 30(1) 27(1) 2(1) 3(1) 0(1)

C(11) 25(1) 27(1) 28(1) -1(1) 4(1) -3(1)

C(12) 21(1) 29(1) 29(1) -2(1) 5(1) -2(1)

C(13) 28(1) 24(1) 32(1) -2(1) 6(1) 2(1)

C(14) 28(1) 31(1) 26(1) -4(1) 6(1) 1(1)

C(15) 23(1) 28(1) 28(1) -1(1) 4(1) -3(1)

C(16) 29(1) 27(1) 30(1) 2(1) 6(1) 0(1)

C(17) 31(1) 29(1) 27(1) -5(1) 6(1) -1(1)

C(18) 22(1) 29(1) 28(1) 1(1) 6(1) -1(1)

C(19) 25(1) 28(1) 32(1) 1(1) 5(1) 0(1)

C(20) 26(1) 27(1) 27(1) 0(1) 4(1) 1(1)

C(21) 40(1) 38(1) 29(1) -6(1) 8(1) 1(1)

C(22) 34(1) 29(1) 25(1) -1(1) 0(1) -2(1)

C(23) 49(1) 41(1) 25(1) 0(1) 0(1) 2(1)

C(24) 29(1) 35(1) 31(1) 5(1) 6(1) 5(1)

C(25) 22(1) 27(1) 28(1) -1(1) 4(1) -2(1)

C(26) 61(1) 48(1) 25(1) 2(1) 7(1) 4(1)

C(27) 38(1) 33(1) 62(1) 12(1) 11(1) 4(1)

C(28) 38(1) 35(1) 41(1) 10(1) 7(1) 4(1)

_______________________________________________________________________
