# Supplementary figures and images for: Crystal structure of 2-butyl­sulfanyl-4,6-bis­[(E)-4-(di­methyl­amino)­styr­yl]pyrimidine
Source: Acta Crystallogr E Crystallogr Commun. 2015 Nov 21;71(Pt 12):o978. doi: 10.1107/S2056989015021441 (PMC4719932; doi:10.1107/S2056989015021441)

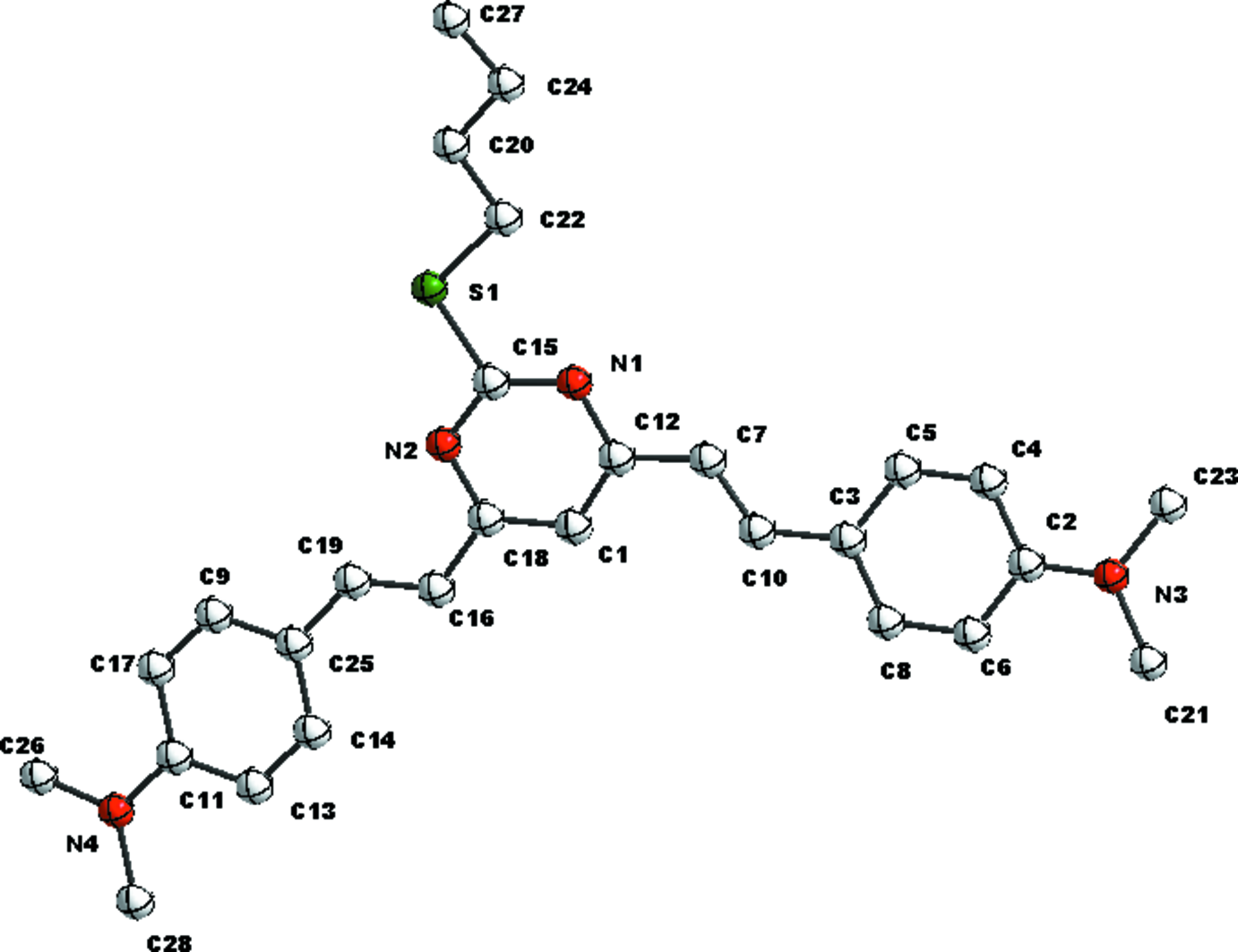

Supplement: Supplementary file 6 [file e-71-0o978-fig1.tif]
